# DMC-LIBSAS: A Laser-Induced Breakdown Spectroscopy Analysis System with Double-Multi Convolutional Neural Network for Accurate Traceability of Chinese Medicinal Materials

**DOI:** 10.3390/s25072104

**Published:** 2025-03-27

**Authors:** Tianhe Huang, Wenhao Bi, Yuxiao Song, Xiaolin Yu, Le Wang, Jing Sun, Chenyu Jiang

**Affiliations:** 1 School of Medical Information Engineering, Shandong University of Traditional Chinese Medicine, Jinan 250355, China; 2022110544@sdutcm.edu.cn (T.H.);; 2 Jinan Guoke Medical Technology Development Co., Ltd., Jinan 250001, China; 3 Jinan Science and Technology Innovation Promotion Center, Jinan 250014, China; 4 Physical Education Department, Shandong University of Traditional Chinese Medicine, Jinan 250355, China; 5 Suzhou Institute of Biomedical Engineering and Technology, Chinese Academy of Sciences, Suzhou 215163, China

**Keywords:** LIBS, CNN, TCM classification, qualitative analysis, origin identification

## Abstract

Against the background of globalization, the circulation range of traditional Chinese medicinal materials is constantly expanding, and the phenomena of mixed origins and counterfeiting are becoming increasingly serious. Tracing the origin of traditional Chinese medicinal materials is of great significance for ensuring their quality, safety, and effectiveness. Laser-induced breakdown spectroscopy (LIBS), as a rapid and non-destructive element analysis technique, can be used for the origin tracing of traditional Chinese medicinal materials. Deep learning can not only handle non-linear relationships but also automatically extract features from high-dimensional data. In this paper, LIBS is combined with deep learning, and a Double-Multi Convolutional Neural Network LIBS Analysis System (DMC-LIBSAS) is proposed for the origin tracing of the traditional Chinese medicinal material *Angelica dahurica*. The system consists of a LIBS signal generation module, a spectral preprocessing module, and an algorithm analysis module—Double-Multi Convolutional Neural Network (DMCNN)—achieving a direct mapping from input data to output results. And the ability of DMCNN to extract characteristic peaks is demonstrated by the 1D Gradient-weighted Class Activation Mapping (1D-Grad-CAM) method. The tracing accuracy of DMC-LIBSAS for *Angelica dahurica* reaches 95.25%. To further verify the effectiveness of the system, it is compared with six classic methods including LeNet, AlexNet, Resnet18, K-nearest neighbors (KNN), Random Forest (RF), and Decision Tree (DT) (with accuracies of 68%, 75%, 72.5%, 79.7%, 86.7%, and 75.5%, respectively), and the tracing effects are all much lower than that of DMC-LIBSAS. The results show that DMC-LIBSAS can effectively and accurately trace the origin of *Angelica dahurica*, providing a new technical support for the quality supervision of traditional Chinese medicinal materials.

## 1. Introduction

As national emphasis on the development of traditional Chinese medicine (TCM) intensifies, markets for TCM formulations, herbs, and medicinal diets have expanded rapidly, leading to a significant increase in demand for medicinal materials [1]. *Angelica dahurica*, a common medicinal herb from the Umbelliferae family, consists of dried roots containing compounds such as imperatorin, angelicin, stearic acid, and sterols. These components exhibit various pharmacological effects, including analgesic properties, cold resistance, nasal congestion relief, swelling reduction, and antioxidant, anti-tumor activities [2]. Variations in growth environments lead to differing levels of active ingredients in *Angelica dahurica* sourced from different regions [3]. Additionally, unique processing techniques contribute to significant price disparities in the market. Given the similar physical appearance of *Angelica dahurica* from various origins, it is challenging to distinguish them visually. Mixing of *Angelica dahurica* from different origins by merchants, whether intentional or inadvertent, can alter the medicinal composition, potentially resulting in ineffective remedies and wasting medicinal resources as well as patients’ time and financial resources. Existing methods for identifying the origin of medicinal materials, including mass spectrometry [4], inductively coupled plasma mass spectrometry [5], and ultraviolet–visible spectrophotometry [6], are constrained by cumbersome pre-treatment procedures, lengthy analysis times, high costs, and complex equipment maintenance. Developing a simple, rapid, and highly accurate method for origin identification is therefore of critical importance.

Laser-induced breakdown spectroscopy (LIBS) is an established analytical technique [7] that utilizes laser pulses to irradiate the surface of a sample, exciting it to a high-temperature plasma state and generating emission spectra. Analysis of characteristic spectral lines of molecules, atoms, and ions in the emission spectra enables the determination of the elemental content of the sample, facilitating both qualitative and quantitative analyses [8,9]. Compared to traditional analytical techniques, LIBS offers significant advantages, including in situ multi-element analysis, remote non-contact detection, minimal sample pre-treatment, rapid detection with minimal material loss, and broad applicability to various sample forms such as solids, liquids, gases, or aerosols [10,11,12]. Recent years have seen an increase in the application of LIBS in origin identification and component detection of medicinal materials. Cai et al. [13] utilized LIBS with an MSC-IGA-SVM machine learning model to trace the origin of *Dioscorea opposita* slices, achieving an identification accuracy of 97.32%. Wang et al. [14] proposed the use of spectral integral normalization and element window filtering for LIBS data normalization and screening, combined with a random forest algorithm for tea origin tracing, confirming the potential of LIBS for tea quality evaluation. Sun et al. [15] integrated LIBS with artificial neural networks to identify *Salvia miltiorrhiza* from different origins through various pre-processing methods, achieving over 95% accuracy in test sets. With advancements in deep learning, the integration of LIBS with deep learning, leveraging its powerful feature extraction and ongoing learning capabilities, has become a research focus in recent years. Pouriyal et al. [16] employed a convolutional neural network combined with long short-term memory methods and LIBS to classify gemstones, validating the approach’s effectiveness and applicability in identification. Gu et al. [17] used multi-view feature extraction convolutional neural networks with LIBS for quantitative analysis of steel alloy elements, providing theoretical foundations and technical support for precise prediction and online quality monitoring. Yan et al. [18] combined LIBS with a multi-scale fusion strategy and proposed a recognition model GAF-DCNN for coal identification, providing a new method for improving the utilization efficiency of coal. Xiao et al. [19] combined multi-scale convolutional neural networks and the ELM method and proposed a method for quickly identifying coal in mining, combustion, and pyrolysis scenarios, with an accuracy rate of 98.3%. Gou et al. [20] proposed an SFEMARNet model that combines channel attention and self-attention mechanisms using LIBS and CNN for soil pollution identification, achieving an accuracy of 98.75% on the test set. Lin et al. [21] combined LIBS with deep learning to identify and diagnose malignant and benign tumors and built an RF-1D model by combining RF and residual networks, with a recognition accuracy rate of 91.1%.

Despite these advancements, systematic methods for origin traceability of Chinese medicinal materials remain limited. To address this gap, this study focuses on *Angelica dahurica* from Anhui and Sichuan provinces, proposing the DMC-LIBSAS. This system integrates a LIBS signal generation module, a spectral preprocessing module, and a Double-Multi CNN(DMCNN) module. The 1D-Grad-CAM method is also applied to verify the performance of DMC-LIBSAS, ensuring its capability to accurately and efficiently determine the origin of *Angelica dahurica*.

## 2. Materials and Methods

### 2.1. DMC-LIBSAS Architecture

**Figure 1:**
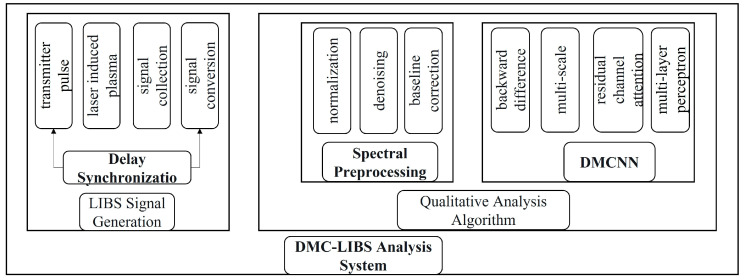
Architecture diagram of the DMC-LIBSAS system.

The proposed DMC-LIBSAS consists of three main modules, as illustrated in Figure 1. The spectral data of *Angelica dahurica* are obtained using the LIBS signal generation device. A laser pulse is directed onto the surface of the *Angelica dahurica* sample, generating plasma. The optical signal emitted by the plasma is collected by the detection head and transmitted to a spectrometer for photoelectric conversion, producing the required spectrum. Under the control of a signal generator, delay synchronization ensures the acquisition of a corresponding spectrum for each laser pulse emission.

The spectral preprocessing module processes the collected spectral data. Through normalization, denoising, and baseline correction algorithms, spectral data with low noise and enhanced characteristic peaks are generated.

The DMCNN module is a custom-designed analysis model tailored for the spectral data of *Angelica dahurica*. The network architecture includes a six-branch CNN with 2 × 3 shared parameters. Incorporating a residual module and channel attention mechanism, the model introduces backward differences to augment input features and multi-scale modules with varying convolution kernel sizes for feature extraction. Prediction results are generated using a multilayer perception (MLP), facilitating an end-to-end learning method that directly maps input data to output results.

These three modules collectively constitute the DMC-LIBSAS, enabling efficient origin traceability for *Angelica dahurica*.

### 2.2. LIBS Spectral Signal Generation Module

**Figure 2:**
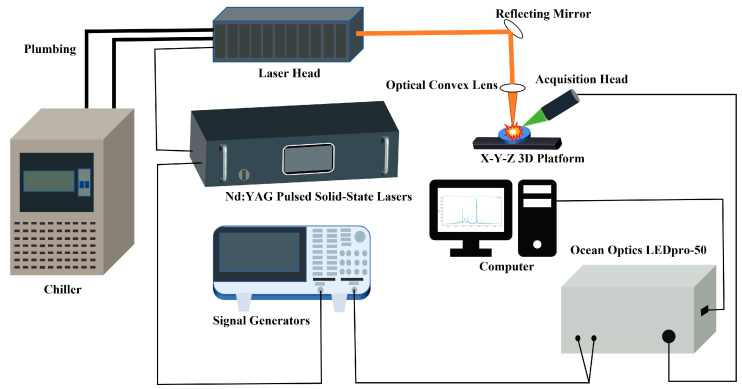
Structural diagram of the LIBS device.

The LIBS experimental setup, depicted in Figure 2, comprises a spectrometer, an Nd:YAG pulsed solid-state laser (Beijing BoXing Keyuan Electronic Technology Co., Ltd., Beijing, China), a laser head (Wuxi LumiSource Technologies Co., Ltd., Wuxi, Jiangsu, China), and a signal controller. Under the control of a signal generator (Tektronix AFG 31000 SERIES, Tektronix Inc., Shanghai, China), trigger signals are sent to a Q-switched Nd:YAG pulsed solid-state laser (custom-built by Harbin Institute of Technology, wavelength: 1064 nm, pulse width: ≤10 ns). Laser pulses generated by the laser head are reflected and focused onto the sample surface using mirrors, resulting in plasma regeneration. Emission signals from the plasma are collected by a probe and transmitted to a spectrometer (Ocean Optics LEDpro-50, spectral range: 370–1100 nm, resolution: 0.8 nm, integrated time: 10 ms, Ocean Optics, Inc., Shanghai, China). Photoelectric conversion, controlled by delay synchronization of the signal controller, produces the LIBS spectra, which are subsequently recorded and displayed on a computer.

### 2.3. Sample Preparation and Data Collection

**Figure 3:**
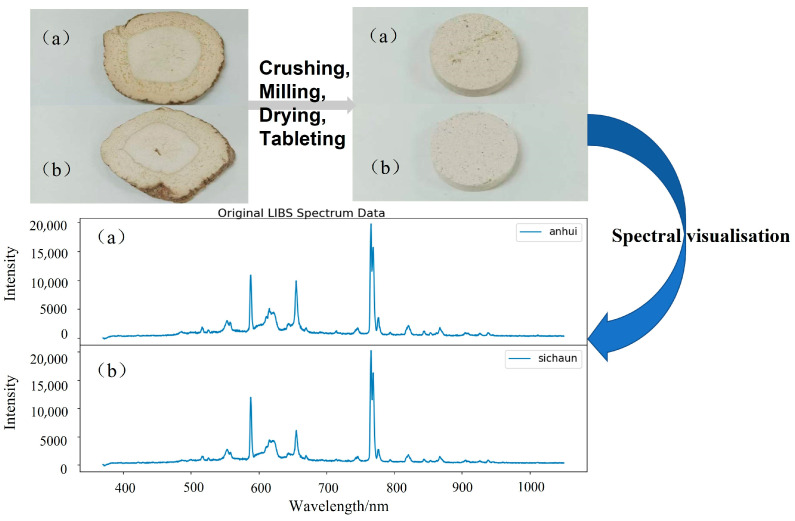
Sample preparation process for *Angelica dahurica*: (**a**) represents samples from Anhui, and (**b**) represents samples from Sichuan.

The *Angelica dahurica* samples used in the experiments were sourced from Anhui and Sichuan provinces, as the medicinal value of *Angelica dahurica* varies significantly depending on its origin. The sample-processing procedure is illustrated in Figure 3, where (a) corresponds to samples from Sichuan, and (b) corresponds to samples from Anhui. The preparation process began with the *Angelica dahurica* samples being ground using a grinder for 2 min, followed by sieving through a 50-mesh sieve. The sieved material was further ground using an agate mortar to obtain a finer powder. The resulting powder was dried in an oven at a constant temperature of 40 °C for 2 h. After drying, the powder was pressed into circular tablets with a diameter of 10 mm under a pressure of 15 Mpa for 3 min. A total of 40 tablets were prepared, with 20 samples from each origin. Optimization of laser parameters revealed that when the laser energy, repetition rate, and delay time were set to 140 mJ, 0.5 Hz, and 2.75 μs, respectively, the resulting spectrum exhibited distinct characteristics with minimal background noise. In the experiment, the peak power density of the pulse reaching the surface of the sample is as high as 7.13 × 10^9^ W/cm^2^, and the ablation diameter is 1 mm. These optimized settings enabled the acquisition of spectral signals with high clarity. For each tablet sample, 50 spectra were collected, resulting in a total of 2000 spectral data points (1000 spectra per origin).

### 2.4. Spectral Preprocessing Module

Denoising, normalization, and baseline correction are essential procedures in spectral data preprocessing.

**Figure 4:**
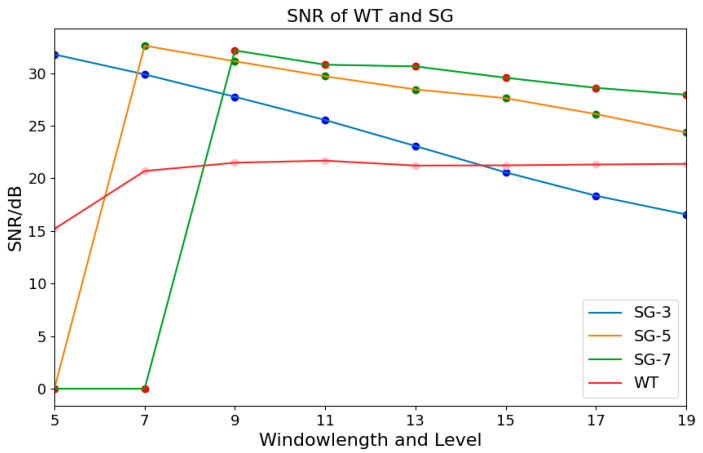
SNR of WT and SG filtering.

In this paper, the data are mapped to the range of [0, 1] by using the max–min normalization. Wavelet Transform (WT) and Savitzky–Golay (SG) [22] filtering are commonly used denoising methods. Linear fitting, polynomial fitting, and polynomial iterative fitting [23] are commonly used baseline correction methods. In order to select the optimal preprocessing method, the signal-to-noise ratio (SNR) and the coefficient of determination are used as the evaluation criteria for denoising and baseline correction. As shown in Figure 4, it presents the SNR of WT and SG filtering. For SG filtering, the fitting orders are set as 3, 5, and 7. The number of decomposition layers for WT ranges from 5 to 19. It is observed that the SNR of SG filtering is generally higher than that of WT. When the fitting order is 5 and the window length is 7, the highest SNR reaches 32.62 dB. Therefore, SG filtering is chosen for denoising.

Table 1 presents the comparison results of the fitting-precision determination coefficients and calculation times of three baseline correction methods. It can be observed that although the polynomial iterative fitting requires the longest calculation time, its fitting precision determination coefficient is the highest, reaching 0.99. Therefore, the polynomial iterative fitting is chosen for baseline correction. In conclusion, this paper adopted max–min normalization, SG filtering, and polynomial iterative fitting for preprocessing the data of *Angelica dahurica* in the experiments.

#### 2.4.1. Savitzky–Golay Filtering

SG filtering smooths data by fitting low-order polynomials to a sliding window of adjacent data points using the least-squares method. This method applies structural dynamic filtering technology to optimize signal processing, effectively retaining the high-order features of the signal while reducing noise. The process involves four key steps.

The first step is polynomial fitting, where a low-order polynomial is applied to the data within the sliding window. The polynomial is expressed by the following formula:

(1) $$y=a_{0}+a_{1}x+a_{2}x^{2}+\ldots +a_{p}x^{p}$$

In this expression, x represents the data to be fitted, y denotes the output data after fitting, a comprises the coefficients to be determined, and p indicates the order of the polynomial, signifying the complexity of the polynomial.

The second step involves calculating the error function, which minimizes the difference between the fitted polynomial and the original data. The error function is expressed as follows:

(2) $$E=\sum_{i=-m}^{m}(y_{i}-(a_{0}+a_{1}x_{i}+a_{2}x_{i}^{2}+\ldots +a_{p}x_{i}^{p}){)}^{2}$$

In this expression, E is the error function used to measure the difference between the fitting polynomial and the original data, m is half the size of the sliding window, so the total window size is *2m* + 1, and $x_{i}$ and $y_{i}$ represent the *i*-th data point within the window and the fitted value corresponding to $x_{i}$, respectively.

The third step calculates the filter coefficients using the following formula:

(3) $$C=(X^{T}X{)}^{-1}X^{T}Y$$

In this formula, *C* denotes the coefficient matrix, *X* is the matrix representation of the polynomial, and *Y* is the matrix form of the original data.

The final step computes the filtered value, as expressed by this formula:

(4) $$y_{k}=\sum_{i=-m}^{m}c_{i}x_{k+i}$$

Here, $x_{k}$ refers to the *k*-th data point in the original data sequence, $y_{k}$ is the *k*-th data point after filtering, *m* represents the order of the filter, and $c_{i}$ indicates the filter coefficient.

#### 2.4.2. Min–Max Normalization

Min–max normalization is a linear transformation method used to map data to a specified interval of [0, 1]. This approach eliminates dimensional differences among different features while preserving the detailed characteristics of the original data. Importantly, it maintains the data’s distribution pattern without altering its overall structure. The normalization process is expressed by the following formula:

(5) $$x^{\prime }=\frac{x-min(X)}{max(X)-min(X)}$$

Here, *x* represents the original value, $x^{\prime }$ denotes the normalized value, and *min*(*X*) and *max*(*X*) refer to the minimum and maximum values in the dataset, respectively. Following this calculation, the normalized value $x^{\prime }$ lies within the interval [0, 1].

#### 2.4.3. Polynomial Iterative Fitting

Polynomial iterative fitting is a commonly used method for baseline correction in spectral data analysis. Building on polynomial fitting, as expressed in Equation (1), this method iteratively fits and refines the original baseline to approximate it more closely with each iteration. Through this iterative process, background noise and interference are effectively reduced or eliminated, improving the accuracy and reliability of spectral analysis.

By employing a combination of SG filtering, min–max normalization, and polynomial iterative fitting, LIBS spectral data with reduced noise and more prominent characteristic peaks can be obtained. These processed spectral data are subsequently used in the DMCNN module for accurate and efficient analysis.

#### 2.4.4. Spectral Preprocessing

**Figure 5:**
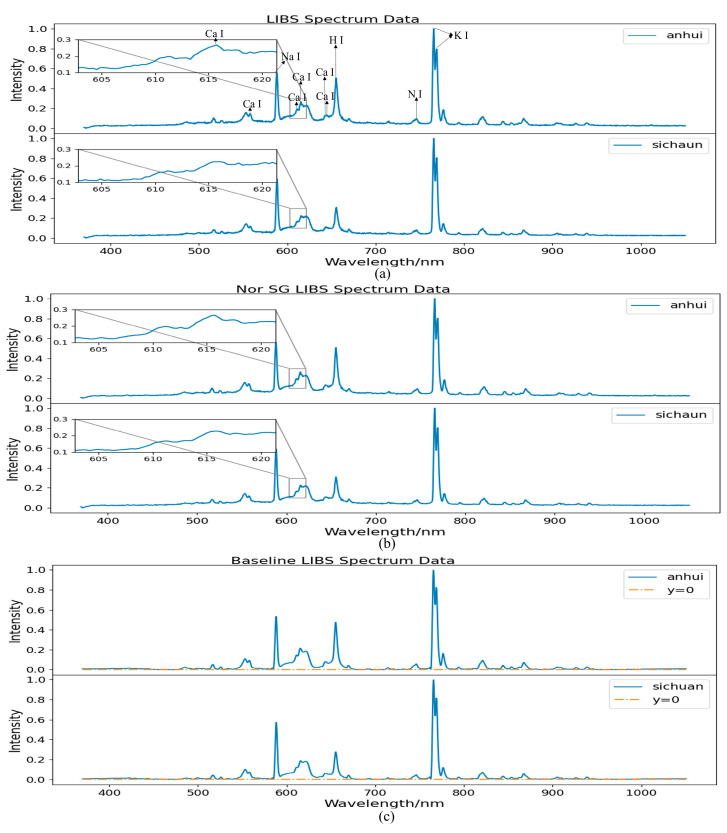
Visualization of the effects of data normalization, SG filtering, and polynomial iterative fitting: (**a**) shows the normalized plot of the original data, (**b**) displays the visualization after SG filtering, and (**c**) presents the visualization following baseline correction.

SG filtering, max–min normalization, and polynomial iterative fitting are commonly used methods for preprocessing spectral data. The effects of these preprocessing steps on the spectral data of *Angelica dahurica* are illustrated in Figure 5. Normalization adjusted the data scale, reduced computational complexity, and enhanced model stability, as shown in (a). SG filtering removed noise, improved the signal-to-noise ratio, and smoothened spectral lines. This improvement is particularly noticeable in the magnified section of the spectral data, as depicted in (b). Polynomial iterative fitting was applied for baseline correction, effectively eliminating the adverse effects of baseline drift on qualitative spectral analysis and improving the quality of the spectral data, as shown in (c). When reviewing the literature of Qasim et al. [24] on the quantitative analysis of *Maerua gromongifolia*, it was found that they not only proposed a correction method utilizing the self-absorption effect in CF-LIBS to extract the relative concentrations of various substances but also marked the spectral lines of multiple elements, such as Ca, Na, N, and K. The characteristic peaks of these elements are also relatively obvious in our spectrum. Therefore, in Figure 5, we have identified the spectral peaks corresponding to the elements Ca, Na, N, and K, and selected one corresponding wavelength for each, which are 616.22 nm, 589.14 nm, 656.26 nm, and 766.49 nm, respectively.

### 2.5. DMCNN Module

#### 2.5.1. Methods for the DNCNN Module

The first-order backward difference method is a numerical differentiation technique used to approximate the derivative of a function at a specific point. In the context of LIBS, the features derived from the backward difference method represent the rate of change in spectral intensity with respect to wavelength. This approach effectively reduces noise and minimizes the influence of background signals, thereby enhancing spectral detail. The method is mathematically expressed as follows:

(6) $$f^{\prime }(x)\approx \frac{f(x)-f(x-h)}{h}$$

Here, *h* is a small positive quantity representing the interval between two adjacent data points, and *f*(*x − h*) denotes the function value at the data point immediately preceding of *x*.

Multi-scale analysis [25] is a technique that captures features across different spatial or temporal scales. This proposed approach employs a parallel multi-branch structure to capture both local and global information, achieving feature fusion and enhancing the model’s expressive capacity.

The residual module addresses challenges such as gradient vanishing and degradation in deep neural network training. By introducing skip connections, the network learns a residual function, thereby improving optimization in deep networks. The residual function is expressed as:

(7) $$y=F(\underline{x,\{Wi\}})+x$$

where *y* denotes the output, *x* is the input, *F*(*x*, {*Wi*}) represents the residual function, and {*Wi*} denotes the network parameters.

The channel attention mechanism focuses on emphasizing important features along the channel dimension by learning the weights of different channel features. This method enhances the network’s response to significant features while reducing attention to less important ones. The process includes three primary operations: Squeeze, Excitation, and Scale. First, the Squeeze operation compresses feature signals to generate a descriptor. Subsequently, a fully connected layer establishes interdependencies among channels; and finally, channel weights adjust sensitivity to features. The process is represented as:

(8) $$z=F_{se}(x)=\sigma (W_{2}Relu(W_{1}x))$$

Here, *x* denotes the input features, $W_{1}$ and $W_{2}$ represent the weight matrices of the fully connected layers, *σ* denotes the Sigmoid activation function, and ReLU represents the Rectified Linear Unit activation function.

#### 2.5.2. Structure of the DNCNN Module

**Figure 6:**
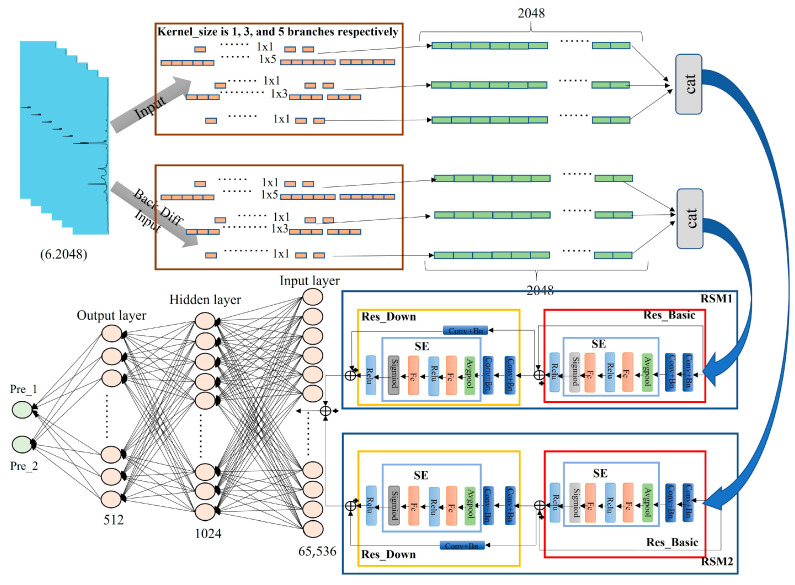
Structural diagram of the DMCNN module.

The core of the system is DMCNN, which aims to rapidly distinguish the origin of *Angelica dahurica* samples. DMCNN is a six-branch CNN with 2 × 3 shared parameters. It combines residual and channel attention mechanisms, introduces backward differences to enhance input features, and adopts multi-scale modules with different convolution kernel sizes for feature extraction. The prediction results are generated by a multi-layer perceptron, achieving a direct mapping from input data to output results. The DMCNN consists of four main components, as shown in Figure 6. The input spectral data size is 6 × 1 × 2048. Initially, backward differencing is applied to form a double branch, augmenting the data and increasing feature input. Subsequently, a three-branch structure is implemented through the multi-scale module, which includes six parallel convolutional layers with activation functions, a max-pooling layer, and an additional convolutional layer. The parallel convolutional layers use 1 × 1, 1 × 3, and 1 × 5 convolution kernels to extract features from the input data. The features extracted by these different convolution kernels are merged to form a unified feature set. The merged features are then processed through the Residual and Channel Attention Module (RSM). This module integrates residual learning and channel attention mechanisms to optimize feature representation. The residual component of the RSM consists of two submodules: Res_Down and Res_Basic, both of which incorporate the SE mechanism. In both submodules, the SE is positioned after the normalization layer and before the activation function. The merged features first pass through Res_Basic, which comprises two convolutional layers, two normalization layers, an SE component, and an activation function. Further feature extraction occurs within the convolutional layers before the features are passed into the SE component. The SE mechanism consists of an average pooling layer and two fully connected layers, performing Squeeze, Excitation, and Scale operations. This process adaptively weights the features of different channels, enhancing attention to critical aspects of the data. Finally, the output features undergo non-linear transformations through an activation function before being forwarded to Res_Down. The Res_Down submodule operates similarly to Res_Basic but incorporates an additional branch with a 1 × 1 convolutional layer. This branch ensures that the input feature dimensions are consistent with those of the main path, facilitating better feature fusion. The outputs of RSM1 and RSM2, which share parameters, are then fused. After passing through these modules, a six-branch convolutional neural network with shared 2 × 3 parameters is constructed. The prediction results are generated through the MLP module. To prevent overfitting, the final dropout layer randomly discards 50% of the network connections during training.

#### 2.5.3. Validation of the DNCNN Module

To verify the performance of the DMCNN model, six classical methods—Random Forest (RF), K-nearest neighbors (KNN), Decision Tree (DT), LeNet, Alexnet, and Resnet18—are employed for comparison, These methods serve as benchmarks to evaluate the effectiveness of the proposed model.

RF, KNN, and DT are widely used machine learning classification algorithms. RF is an ensemble learning method that improves model accuracy by constructing multiple decision trees and aggregating their predictions. KNN classifies samples by calculating the distance between features and selecting the K-nearest neighbors. DT builds decision rules by recursively partitioning the dataset into smaller subsets for classification. Despite their popularity, these methods exhibit high computational complexity and are sensitive to noise, necessitating careful parameter tuning. To ensure model performance, five-fold cross-validation is used to evaluate the model’s accuracy.

LeNet, AlexNet, and ResNet18 are commonly used classical deep learning methods. LeNet, a pioneering convolutional neural network, consists of convolutional, pooling, and full connected layers. AlexNet introduced the ReLU activation function within the convolutional networks and successfully applied the dropout technique, addressing issues such as overfitting and gradient dispersion in deep learning. ResNet18 innovatively introduced residual connections, a key innovation that effectively mitigates gradient vanishing and degradation problems in deep neural networks.

All comparison methods are evaluated using accuracy as the primary metric, which is calculated as follows:

(9) $$Accuracy=\frac{TP+TN}{TP+TN+FP+FN}$$

where TP, TN, FP, and FN represent true positives, true negatives, false positives, and false negatives, respectively. This comparative analysis ensures a comprehensive evaluation of the DMCNN model’s performance relative to established machine learning and deep learning approaches.

## 3. Results

This chapter presents an experimental validation of the performance of the DMC-LIBSAS. Using samples of *Angelica dahurica* from Anhui and Sichuan provinces, the system’s performance is evaluated based on sample recognition accuracy, visualization analysis, ablation experiments, and comparisons with multiple models. The experimental results are obtained through the DMCNN module during the data analysis process, and then the performance of the DMCNN module in the DMC-LIBSAS for data traceability is verified through ablation experiments and comparative experiments. The code in this paper was completed on PyCharm. The experimental code is available for use, and the source code will be uploaded to GitHub later.

### 3.1. Model Performance Validation

The CNNs automatically extract features, filter out irrelevant information, and map these features into high-dimensional spaces to form rich feature representations. Building on this foundation, CNNs progressively extract more advanced and abstract features. The DMCNN proposed in this study was built on PyTorch version 1.8, using CrossEntropyLoss as the loss function and SGD as the optimizer. The learning rate was set to 0.00001, and training was conducted over 300 iterations. The normalized dataset was divided into training, validation, and test sets in a 6:2:2 ratio, resulting in sample sizes of 1200, 400, and 400, respectively.

**Figure 7:**
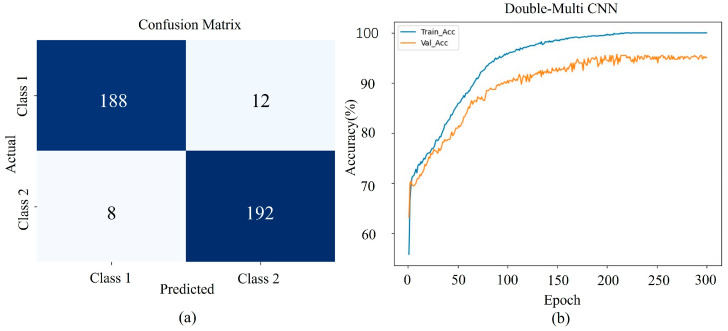
DMCNN prediction results and visualizations: (**a**) shows the confusion matrix, and (**b**) presents a visualization of the training and validation sets with training results across epoch iteration.

Figure 7 illustrates the performance of the six-branch DMCNN with 2 × 3 shared parameters: (a) presents the confusion matrix for the prediction results, while (b) visualizes the training and validation set performance across epoch iterations. The network achieved a classification accuracy of 95.25% on the test set using *Angelica dahurica* data. In the confusion matrix, Class 1 corresponds to samples from Anhui, and Class 2 corresponds to samples from Sichuan. The number of correctly predicted samples in the test set was 188 for the Anhui production area and 192 for the Sichuan production area. To further demonstrate the model’s performance on the training and validation sets, the prediction results were saved and visualized as shown in Figure 7b. Around 225 epochs, the accuracy of both the training and validation sets began to stabilize, indicating consistent model performance and effective learning throughout the training process.

**Figure 8:**
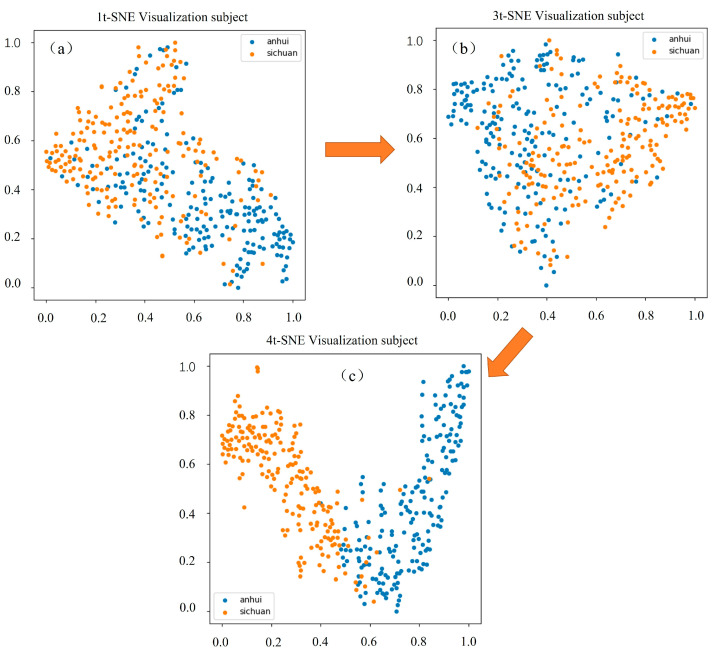
t-SNE visualization of output features for (**a**) the multiscale module, (**b**) the residual channel attention module, and (**c**) the final fully connected layer in DMCNN.

Figure 8 presents the t-distributed Stochastic Neighbor Embedding (t-SNE) dimensionality reduction visualization results of the output features from the multi-scale module, residual channel attention module, and the final fully connected layer in the DMCNN. The visualization began with panel (a), which represents the features extracted by the parallel multi-branch structure, and progressed to panel (b), depicting the residual channel attention module. The results in panels (a) and (b) indicate that the boundaries between the production areas of *Angelica dahurica* are not clearly defined, indicating that the features extracted by these shallower layers are less distinctive for differentiating between production areas. In panel (c), the visualization of the features from the final fully connected layer reveals a well-defined boundary separating the production areas. This observation demonstrates that the deepest layer of the network has effectively learned representative features that distinguish the *Angelica dahurica* samples based on their origin.

**Figure 9:**
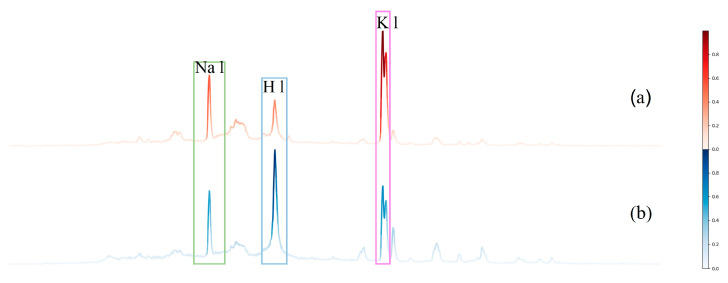
1D-Grad-CAM-like activation visualization: (**a**) represents *Angelica dahurica* samples from Anhui, while (**b**) represents *Angelica dahurica* samples from Sichuan.

Deep learning is often regarded as a “black box” due to the complexity of its internal structure and the absence of explicit causal relationships [26]. To enhance the interpretability of the predictive decisions made by the DMCNN, the 1D-Grad-CAM method was employed to visualize the class activations of key regions learned by the model. The results are shown in Figure 9, where panel (a) corresponds to *Angelica dahurica* from Anhui (colored in red), and panel (b) corresponds to *Angelica dahurica* from Sichuan (colored in blue). The intensity of the color represents the importance of the region, with darker colors indicating higher relevance. In panel (a), the region within the pink box exhibits the darkest color, signifying its importance in the classification of Anhui-origin *Angelica dahurica*. Similarly, in panel (b), the region within the blue box shows the darkest color, indicating its critical role in identifying Sichuan-origin *Angelica dahurica*. Other elemental characteristic peaks, such as those in the green box, were also extracted, further demonstrating the model’s capability to abstract deep information and identify features corresponding to specific elemental characteristic peaks. These findings suggest that the DMCNN effectively captures and utilizes deep spectral information, enabling more precise qualitative analysis of *Angelica dahurica* based on its origin.

### 3.2. Ablation Experiments

**Figure 10:**
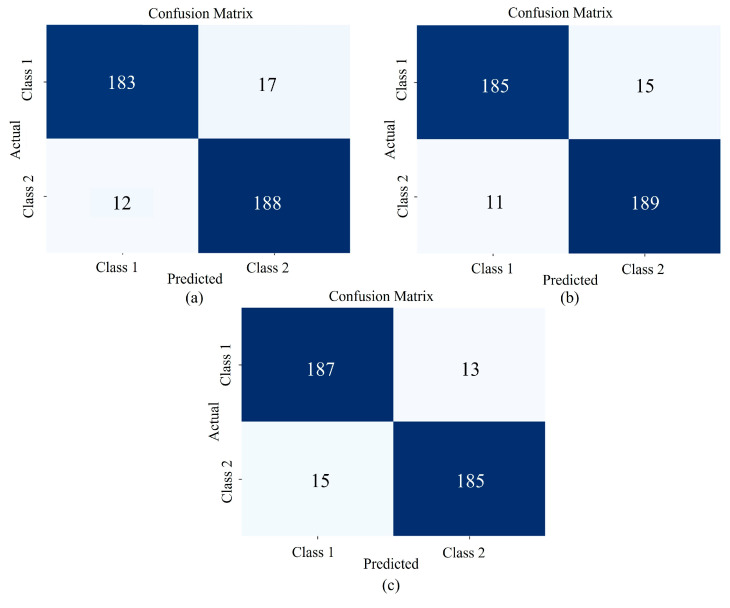
Confusion matrix corresponding to the results of each module in the ablation experiment: (**a**) represents the confusion matrix for the residual channel attention module, (**b**) represents the confusion matrix with the addition of backward differencing, and (**c**) represents the confusion matrix with the addition of multiscaling.

To verify the performance of each component in the DMCNN, ablation experiments were conducted. Predictions were performed on the residual and channel attention base module, the base module with the inclusion of backward difference, and the base module with the introduction of multi-scale features. The confusion matrix results are shown in Figure 10, where Class 1 represents *Angelica dahurica* from Anhui and Class 2 represents *Angelica dahurica* from Sichuan. The accuracy of each module on the training set (Tra), validation set (Val), and test set (Pre) is summarized in Table 2. Panel (a) in Figure 9 displays the prediction results for the residual and channel attention base module, which achieved an accuracy of 92.75% on the test set. Panel (b) shows the prediction results after incorporating the backward difference method into the base module, improving test set accuracy to 93.5%. Panel (c) illustrates the prediction results after introducing the multi-scale module into the base module, achieving a test set accuracy of 93.25%. These results indicate that the six-branch DMCNN with 2 × 3 shared parameters achieves the highest accuracy in discriminating the production area of *Angelica dahurica*. The addition of the backward difference and multi-scale modules significantly improves the model’s performance compared to the residual and channel attention base model alone.

### 3.3. Comparative Experiments

**Figure 11:**
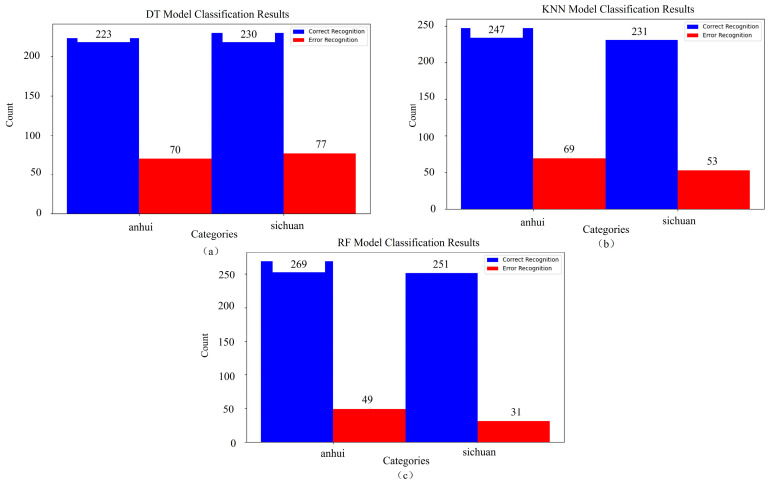
Classification results of machine learning methods: (**a**) visualization of classification results for DT, (**b**) visualization of classification results for KNN, and (**c**) visualization of classification results for RF.

The classification performance of three machine learning methods—RF, KNN, and DT—was compared. Table 3 summarizes the parameter settings and prediction results of these methods. The dataset was divided into a training set and a validation set in a 7:3 ratio, resulting in 1400 and 600 samples, respectively. Figure 11 shows the results for TP, TN, FP, and FN, where blue indicates correct classifications and red indicates incorrect classifications. Panel (a) displays the classification results for DT, with correct classifications for Anhui and Sichuan being 223 and 230, respectively, and achieving a five-fold cross-validation accuracy of 75.5%. Panel (b) shows the classification results for KNN, with correct classifications of 247 and 231, respectively, and achieving a five-fold cross-validation accuracy of 79.7%. Panel (c) presents the classification results for RF, with correct classifications of 269 and 251, respectively, and achieves the highest five-fold cross-validation accuracy of 86.7%. These results highlight the superior performance of RF compared to KNN and DT in accurately classifying the origin of *Angelica dahurica*.

**Figure 12:**
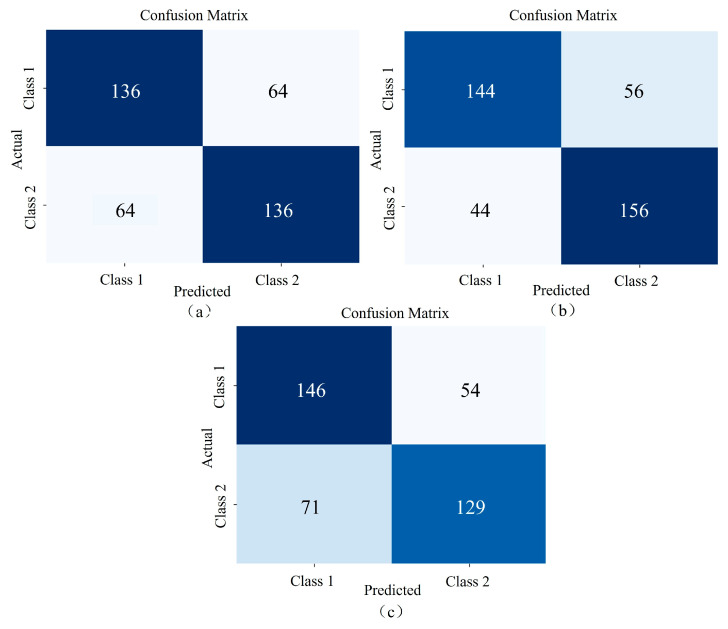
Confusion matrices comparing the prediction results of classical network structures: (**a**) represents LeNet, (**b**) represents the improved AlexNet, and (**c**) represents Resnet18.

LeNet, AlexNet, and ResNet18 are commonly used classical CNNs. To better accommodate the high-dimensional nature of spectral data, modifications were made to the original AlexNet model. Specifically, MaxPool1d layers in the first two layers of the network were replaced with BatchNorm1d. This adjustment was designed to prevent premature dimensionality reduction, which could lead to the loss of important features, while maintaining the remaining structure of the model unchanged. The dataset was divided into training, validation, and test sets in a 6:2:2 ratio. Table 4 shows the classification accuracy of LeNet, the modified AlexNet, and Resnet18 on the dataset. Figure 12 illustrates the confusion matrix for the test set, where Class 1 corresponds to *Angelica dahurica* from Anhui, and Class 2 corresponds to *Angelica dahurica* from Sichuan. Panel (a) shows the confusion matrix for LeNet, with 136 correctly classified samples for both Anhui and Sichuan, resulting in an accuracy of 68%. Panel (b) shows the confusion matrix for the modified AlexNet, with 144 correctly classified Anhui samples and 156 correctly classified Sichuan samples, achieving an accuracy of 75%. Panel (c) presents the confusion matrix for ResNet18, with 151 correctly classified Anhui samples and 139 correctly classified Sichuan samples, yielding an accuracy of 72.50%.

Compared to the six methods discussed above, the DMCNN module achieved the highest accuracy in classifying *Angelica dahurica* data. By incorporating backward differences to increase input features and conducting parallel multi-scale feature extraction, the module leverages residual and channel attention mechanisms to overcome the issue of gradient vanishing. This approach enables the effective extraction of characteristic peak information, significantly enhancing the performance of the DMC-LIBSAS.

**Figure 13:**
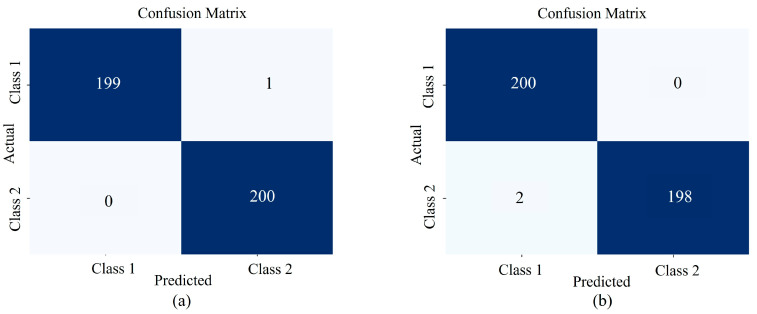
The confusion matrix of DMC-LIBSAS for the classification results of *Fritillaria cirrhosa* (**a**) and *Glycyrrhiza uralensis* (**b**).

To verify the effectiveness of DMC-LIBSAS, the origin tracing of *Fritillaria cirrhosa* (from Yunnan and Zhejiang) and *Glycyrrhiza uralensis* (from Gansu and Inner Mongolia) was subsequently conducted. Under the same experimental conditions and system parameters, the accuracy rates of traceability were 99.75% and 99.5%, respectively. Figure 13 below shows the confusion matrix of the classification results. The results indicate that this system has a high precision in tracing the origin of Chinese herbal medicines.

## 4. Conclusions

This study elaborates on the DMC-LIBSAS, which integrates data collection, processing, and analysis into an end-to-end workflow, enabling accurate and efficient origin traceability of *Angelica dahurica* from Anhui and Sichuan provinces. At the core of the system is the DMCNN module, a six-branch CNN with 2 × 3 shared parameters, incorporating residual and channel attention mechanisms, backward differences, and multi-scale feature extraction. Prediction results are generated through a multi-layer perceptron, achieving a prediction accuracy of 95.25%. Both comparative and ablation experiments demonstrate that the DMC-LIBSAS, powered by the DMCNN module, achieves optimal performance. Additionally, the feature extraction capability of the DMCNN module was verified using the 1D-Grad-CAM method, which highlights its ability to accurately identify characteristic peaks of relevant elements. These findings confirm the precision, efficiency, and robustness of the DMC-LIBSAS for origin traceability tasks.

## Figures and Tables

**Table 1:** The fitting-precision determination coefficients and computational times of the three baseline correction methods.

Methods	Decision Coefficient for Fitting Accuracy	Computational Time
SG–Normalization–Polynomial Iterative Fitting	0.99	0.009
SG–Normalization–Polynomial Fitting	0.09	0.004
SG–Normalization–Linear Fitting	0.05	0

**Table 2:** Performance metrics of each module in the ablation experiment.

Model	Tra (%)	Val (%)	Pre (%)
Base Module	100.00	92.50	92.75
Introduction of backward difference	100.00	95.00	93.50
Introduction of multiscale	100.00	94.75	93.25

**Table 3:** Parameter settings and accuracy metrics for RF, KNN, and DT.

Machine Learning Method	Main Parameter	Test Accuracy (%)
KNN	n_neighbors = 5	79.7
RF	n_estimators = 100, random_state = 42	86.7
DT	random_state = 42	75.5

**Table 4:** Prediction results for training, validation, and testing phases of LeNet and the improved AlexNet.

Model	Tra (%)	Val (%)	Pre (%)
LeNet	71.5	70	68
AlexNet	73.25	73.75	75
ResNet18	100%	79%	72.50%

## Data Availability

The original contributions presented in this study are included in the article. Further inquiries can be directed to the corresponding author(s).

## Abbreviations

The following abbreviations are used in this manuscript:

LIBS	Laser-induced Breakdown Spectroscopy
DMC-LIBSAS	Double-Multi Convolutional Neural Network LIBS Analysis System
KNN	K-Nearest Neighbors
RF	Random Forest
DT	Decision Tree
DMCNN	Double-Multi Convolutional Neural Network
WT	Wavelet Transform
SNR	Signal-to-Noise Ratio
1D-Grad-CAM	1D Gradient-weighted Class Activation Mapping
TCM	Traditional Chinese Medicine
MLP	Multi-Layer Perceptron
SG	Savitzky–Golay
RSM	Residual and Channel Attention Module
CNN	Convolutional Neural Network
Tra	Training Set
Val	Validation Set
Pre	Test Set
t-SNE	t-distributed Stochastic Neighbor Embedding
